# The key cellular senescence related molecule RRM2 regulates prostate cancer progression and resistance to docetaxel treatment

**DOI:** 10.1186/s13578-023-01157-6

**Published:** 2023-11-15

**Authors:** Bisheng Cheng, Lingfeng Li, Yongxin Wu, Tianlong Luo, Chen Tang, Qiong Wang, Qianghua Zhou, Jilin Wu, Yiming Lai, Dingjun Zhu, Tao Du, Hai Huang

**Affiliations:** 1 Department of Urology, Sun Yat-Sen Memorial Hospital, Sun Yat-Sen University, Guangzhou, 510120 China; 2 Guangdong Provincial Key Laboratory of Malignant Tumor Epigenetics and Gene Regulation, Sun Yat-Sen Memorial Hospital, Sun Yat-Sen University, Guangzhou, 510120 China; 3 Guangdong Provincial Clinical Research Center for Urological Diseases, Sun Yat-Sen Memorial Hospital, Sun Yat-Sen University, Guangzhou, 510120 China; 4 Department of Urology, The Sixth Affiliated Hospital of Guangzhou Medical University, Qingyuan People’s Hospital, Qingyuan, 511518 Guangdong China; 5 Department of Urology, Nanfang Hospital, Southern Medical University, Guangzhou, 511430 China; 6 Department of Obstetrics and Gynecology, Sun Yat-Sen Memorial Hospital, Sun Yat-Sen University, Guangzhou, 510120 Guangdong China

**Keywords:** RRM2, Prostate cancer, Chemotherapy, Docetaxel, Oncology

## Abstract

**Background:**

Prostate cancer is a leading cause of cancer-related deaths among men worldwide. Docetaxel chemotherapy has proven effective in improving overall survival in patients with castration-resistant prostate cancer (CRPC), but drug resistance remains a considerable clinical challenge.

**Methods:**

We explored the role of Ribonucleotide reductase subunit M2 (RRM2), a gene associated with senescence, in the sensitivity of prostate cancer to docetaxel. We evaluated the RRM2 expression, docetaxel resistance, and ANXA1 expression in prostate cancer cell lines and tumour xenografts models. In addition, We assessed the impact of RRM2 knockdown, ANXA1 over-expression, and PI3K/AKT pathway inhibition on the sensitivity of prostate cancer cells to docetaxel. Furthermore, we assessed the sensitivity of prostate cancer cells to the combination treatment of COH29 and docetaxel.

**Results:**

Our results demonstrated a positive association between RRM2 expression and docetaxel resistance in prostate cancer cell lines and tumor xenograft models. Knockdown of RRM2 increased the sensitivity of prostate cancer cells to docetaxel, suggesting its role in mediating resistance. Furthermore, we observed that RRM2 stabilizes the expression of ANXA1, which in turn activates the PI3K/AKT pathway and contributes to docetaxel resistance. Importantly, we found that the combination treatment of COH29 and docetaxel resulted in a synergistic effect, further augmenting the sensitivity of prostate cancer cells to docetaxel.

**Conclusion:**

Our findings suggest that RRM2 regulates docetaxel resistance in prostate cancer by stabilizing ANXA1-mediated activation of the PI3K/AKT pathway. Targeting RRM2 or ANXA1 may offer a promising therapeutic strategy to overcome docetaxel resistance in prostate cancer.

**Graphical Abstract:**

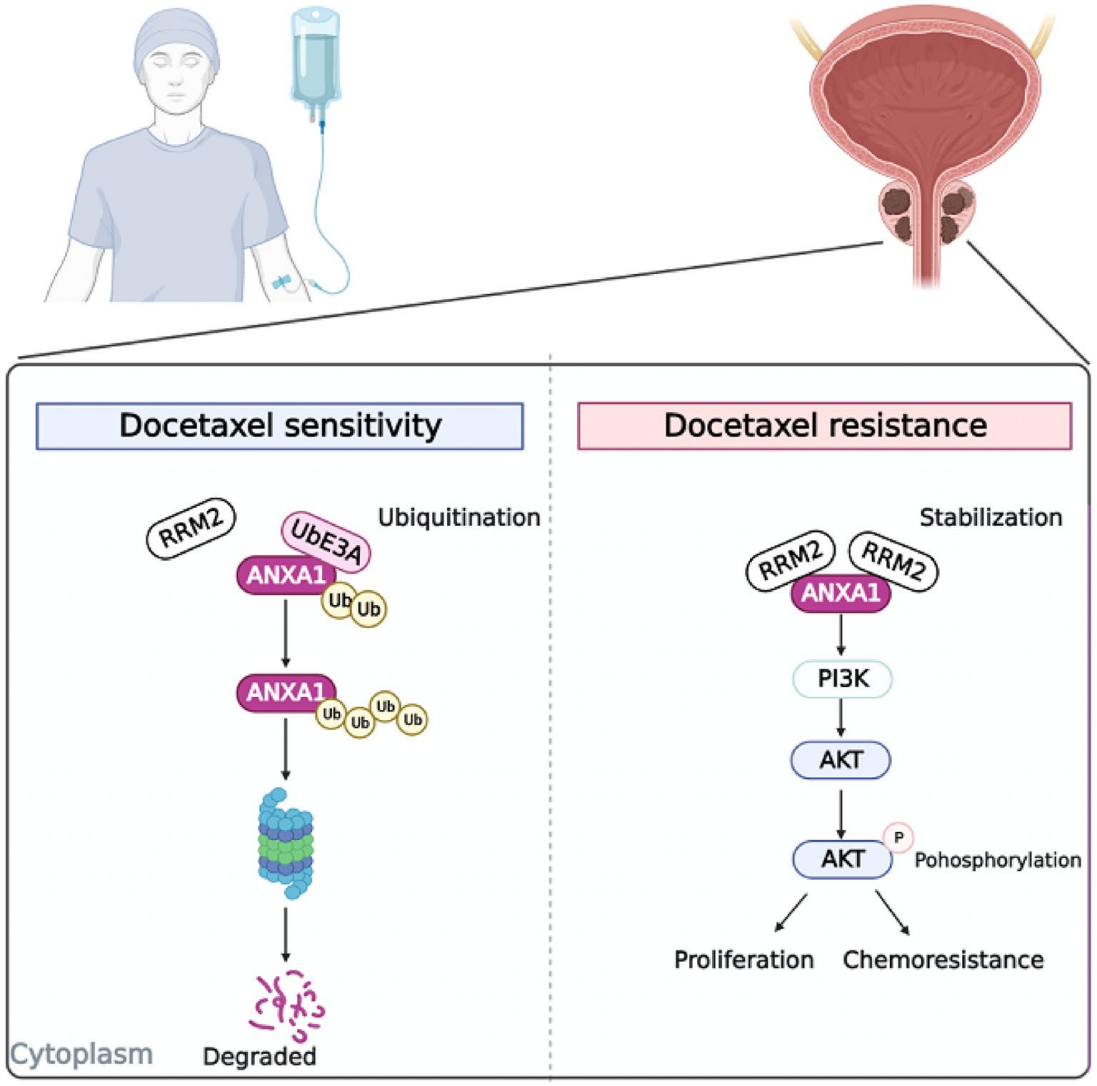

**Supplementary Information:**

The online version contains supplementary material available at 10.1186/s13578-023-01157-6.

## Introduction

Prostate cancer is the most commonly diagnosed cancer in men and a leading cause of cancer-related death worldwide [1]. Androgen deprivation therapy (ADT) is the widely used therapy method for PCA, but there exists resistance problem in most cases, and caused castration-resistant prostate cancer (CRPC) [2]. Docetaxel is a chemotherapeutic agent that has been shown to improve overall survival in patients with CRPC [3, 4]. However, not all patients respond to docetaxel, and those who do respond eventually develop resistance [5]. Therefore, there is a need to identify new therapeutic targets and improve the efficacy of docetaxel therapy in prostate cancer.

Cellular senescence is a state of permanent growth arrest that is induced in response to various stresses, including DNA damage and oncogene activation [6]. Senescence is thought to play a role in cancer development and progression, and recent studies have shown that senescence-related genes may be involved in regulating sensitivity to cancer therapies [7–9]. Ribonucleotide reductase subunit M2 (RRM2) is a key enzyme in the synthesis of deoxyribonucleotides and is essential for DNA synthesis and repair [10]. RRM2 has been shown to be upregulated in various types of cancer, including prostate cancer, and is associated with poor prognosis and resistance to chemotherapy [11, 12]. However, the impact of RRM2 on the sensitivity of PCA to docetaxel treatment is not yet clear.Annexin A1 (ANXA1) is a calcium dependent phospholipid binding protein closely related to various cellular activities, such as inflammation and apoptosis, as well as cancer cell proliferation [13, 14]. Related experimental studies have found that ANXA1 is upregulated in various cancer tissues, including prostate cancer, and is associated with chemotherapy resistance. Its high expression generally indicates poor prognosis [15–17]. ANXA1 has also been shown to activate the PI3K/AKT pathway, a key signalling pathway that regulates cell proliferation and survival [15–17]. However, the role of ANXA1 in regulating sensitivity to docetaxel therapy in prostate cancer is not well understood.

In this study,we investigated the roles of RRM2 and the ANXA1 in regulating sensitivity to docetaxel therapy in prostate cancer. Additionally,we explored the potential for targeting RRM2 and the ANXA1 as a therapeutic strategy to enhance the efficacy of docetaxel therapy.

## Methods

### TCGA, GEO and PCaDB genomics platform data mining

Patient clinical data from the TCGA Prostate Adenocarcinoma (PRAD) cohort were obtained from the official website of the National Cancer Institute’s Cancer Genome Atlas (TCGA) [18]. Kaplan–Meier survival analysis, which assessed survival outcomes based on RRM2 (Ribonucleotide Reductase Regulatory Subunit M2) expression levels, and the association between RRM2 and ANXA1 (Annexin A1) expression levels within this cohort, were downloaded from the Gene Expression Profiling Interactive Analysis (GEPIA) platform [19], which can be accessed at http://gepia.cancer-pku.cn/index.html. Additionally, various transcriptome sequencing datasets, with different case grouping methods, were gathered from the Gene Expression Omnibus (GEO) repository [https://www.ncbi.nlm.nih.gov/geo/]. These datasets were used for further analyses.The results of an integrated analysis, incorporating prognostic information from multiple databases, can be accessed on the Prostate Cancer Database (PCaDB) website [http://bioinfo.jialab-ucr.org/PCaDB/]. This comprehensive resource provides valuable insights into prostate cancer prognosis and related information derived from a multitude of data sources.

### Cell culture and transfection

The prostate cancer cell lines PC-3, DU145, LNCaP, and 22Rv1 were procured from the American Type Culture Collection (ATCC), situated in Massachusetts, Virginia, USA. These cell lines were cultured in RPMI-1640 medium (Gibco, USA, Catalog Number 1640-C11875500BT) supplemented with 10% fetal bovine serum (FBS) obtained from Gibco (Catalog Number 10099141). Cell incubation was conducted under controlled conditions at 37 ℃ in an environment enriched with 5% CO_2_. Transient transfection was facilitated using Lipofectamine 2000 (Thermo Fisher Scientific, Catalog Number 11668019).

For RNA interference experiments, we utilized small interfering RNAs (siRNAs) targeting specific genes, including RRM2, ANXA1, as well as a negative control siRNA. These siRNAs were procured from IGE Biotechnology Ltd (China) and their details can be found in Additional file 1: Table S2. The siRNA transfections were executed following the manufacturer’s guidelines and previously established protocols [20]. Briefly, a mixture containing 5 μL of dissolved siRNA and 3 μL of Lipofectamine RNAiMAX (Invitrogen, Carlsbad, California, USA) in 200 μL of OPTI‐MEM (Gibco, Carlsbad, California, USA) was prepared at 25 ℃ for 20 min. Subsequently, this mixture was added to the cells and incubated for a duration of 48 h.Transient transfections, lentivirus production, and cell infections were conducted as described previously [21]. Briefly, plasmids were mixed with X‐tremeGENE (Invitrogen) at 25 ℃ for 20 min. Following this incubation, the mixture was added to the cells and incubated for 24–48 h. To generate lentivirus, HEK‐293 T cells were transfected with psPAX2 and PMD2.G plasmids (both from IGE) in conjunction with the stably silenced or overexpressed vectors, using X‐tremeGENE. After a 48-h incubation, lentiviruses were harvested, filtered, and concentrated. Subsequently, cells were infected with these lentiviruses using polybrene (IGE) and then subjected to puromycin-based selection.

### Cell viability assay

The assay was performed as the previous study [22]. In this experimental study, we assessed cell viability using a Cell Counting Kit-8 (CCK-8, obtained from APExBIO, Catalog Number K1018), following the manufacturer’s provided instructions meticulously. To carry out this assessment, cells were carefully seeded in 96-well plates and subjected to various concentrations of docetaxel treatment for a duration of 48 h. After this treatment period, the CCK-8 reagent was added to each well, and the cells were incubated for an additional 2 h.The optical density (OD) at 450 nm was subsequently measured using a dedicated photometer (BioTek), allowing us to quantitatively evaluate cell viability and assess the impact of docetaxel treatment on the cells under investigation.

### Apoptosis assay

The assay was operated as previously mentioned [23].Apoptosis was measured based on Annexin V-FITC Kit (Elabscience E-CK-A211-100) following the manufacturer’s protocol. To initiate the cellular response, cells were cultured in docetaxel-containing medium in 6-well plates for 48 h. The treated cells were harvested, washed with PBS, and suspended in Annexin V-FITC- and PI buffer for 15 min in the dark. Subsequently, apoptotic cells were detected through flow cytometric analysis using the Apoptosis Detection Kit (Beckman cytoFLEX, USA).

### Western blot analysis

Proteins in samples were separated based on SDS–PAGE and transferred onto nitrocellulose membranes (Bio-Rad. Subsequently, 5% nonfat milk in Tris-buffered saline was added to the membranes, and cultured with primary antibodies for 12 h at 4 ℃.After that, it was incubated with horseradish peroxidase-conjugated secondary antibodies at 25 ℃ for 1 h. To obtain the visualized result, we used an enhanced chemiluminescence (ECL) detection system from Proteintech (PK10003).

### Coimmunoprecipitation (Co–IP) and mass spectrometry (MS) analysis

Co-IP was conducted as illustrated in the former publication [24]. The interaction between RRM2 and ANXA1 was detected in wild-type PC3 and DU145 cells. In short, nuclear extracts were cultured with anti-RRM2, anti-ANXA1 at 4 ℃for 16 h and then treated via protein A/G magnetic beads for 2 h at room temperature. Immunoreactive proteins were measured based on WB method. MS testing was carried out at the Bioinformatics and Omics Center in our Hospital.

### Quantitative real-time PCR (qPCR)

Total RNA was extracted via TRIzol (Thermo, 15596026) according to the relevant instrument. To synthesize cDNA, the HiScript II One Step RT‒PCR Kit (P611-01) was used. The qPCR was performed based on SYBR Green Mix (Vazyme, Q711-02). The primers applied in qPCR were exhibits in Additional file 1: Table S2. The 2^^−ΔΔ^Ct method were applied to calculate the relative mRNA levle, and the level were normalized based on GAPDH.

### SA-β-gal staining assay

Culture the PC3 and DU145 cells in appropriate cell culture medium (RPMI-1640, DMEM, respectively) supplemented with 10% fetal bovine serum (FBS) and antibiotics.Treat the cells with DTX at the IC50 concentration for 24 h. Ensure to prepare a range of DTX concentrations around the IC50 value to generate a dose–response curve.Fix the cells: a. Remove the culture medium and wash the cells with PBS. b. Fix the cells using 4% paraformaldehyde for 10–15 min at room temperature. Continue with steps 3–5 mentioned for SCI samples to perform the SA-β-gal staining assay.Wash: a. Rinse the fixed cells with phosphate-buffered saline (PBS) twice to remove any residual fixative.Preparing the staining solution: a. Follow the manufacturer’s instructions of the SA-β-gal staining kit to prepare the staining solution. b. Typically,the staining solution contains X-gal (5-bromo-4-chloro-3-indolyl-β-D-galactoside) as a substrate for β-galactosidase activity detection. c. Adjust the pH of the staining solution based on the kit instructions (PH = 6). Staining: a. Incubate the fixed tissue or sections with the staining solution at 37 ℃ for the recommended incubation time mentioned in the kit instructions. b. Monitor the staining process under a microscope for the development of a blue color, indicating the presence of β-galactosidase activity (Abbkine, E-CK-A211-100, KTA3030).

### Ubiquitination assay

Cells were transfected with siCtrl for 48 h and treated by inhibitor MG132 (InvivoGen, tlrl-mg132), for 6 h. Following treatment, the cells were harvested, lysed in RIPA buffer containing a protease inhibitor cocktail, and then sonicated. The lysates were incubated with IgG control for 16 h at 4 ℃ and subsequenly cultured with A/G-agarose beads (Thermo, USA) for 2 h at 4 ℃. The resulting immunoprecipitates were then washed with RIPA buffer and subjected to Western blot analysis.

### Immunofluorescence (IF) staining

IF staining was performed according to the methods described in former research [25]. The PCa cells were put into confocal dishes, fixed and prehybridized with 0.5% Triton X–100. Subsequently, they were blocked and cultured with anti-RRM2 and anti‐ANXA1 at 4 ℃ for 15 h. And then, the dishes were washed via PBS and cultured with secondary antibodies at room temperature for 1 h. Subsequently, incubated with DAPI (Solarbio) for 5 min at room temperature. The confocal microscope (Zeiss, Germany) was applied to obtain the images.

### ELISA‐based quantification of secreted IL-6 and IL-8

The cell culture supernatant was collected, and the secreted IL-6/IL-8 was quantified using Human IL-6/IL-8 ELISA Kit (MEIMIAN,MM-0049H2;MM-1558H2,China) according to the manufacturer’s instructions.Briefly, the supernate from PCa cells was collected, diluted at 1:2, and added into the wells coated with IL-6/IL-8 antibody.After incubation in a 37 ℃ incubator for 30 min, the absorbance of each well at 450 nm was measured. We then calculated each well's content of IL-6/IL-8 according to the standard curve.

### Immunohistochemistry (IHC)

IHC was carried out according to previous research [26, 27]. In the experimental process, the sample was dewaxed, rehydrated, and cultured with protease K at 37 ℃ for 15 min. Then, it was cultured with 3% H_2_O_2_ for 10 min at 25 ℃ to inhibit peroxidase activity. Subsequently, the sample was incubated with primary antibodies for 15 h at 4 ℃. After washed via PBS, it was cultured with biotinylated secondary antibodies for 1 h at 25 ℃, followed by staining with DAB solutions (ZSGB–BIO, China). And then washed and counterstained with HE. IHC analyses were perform according to former described. The standard scoring for this test involves grading the staining intensity of cells on a scale of 0 to 4, where a score of 0 corresponds to no positive staining (negative), a score of 1 corresponds to weak positive staining (light yellow), a score of 2 corresponds to positive staining (brownish-yellow), and a score of 3 corresponds to strong positive staining (brown). In addition, the score of positive cells is determined based on the percentage, with percentage below 25% mark 1 score; 26–50% and 51%–75% mark 2 and 3 score respectively; above 75%mark 4 score. The final score was determined based on multiply the dyeing intensity score by the dyeing ratio score. Cell staining images were collected based on the Nikon ECLIPSE system (Tokyo, Japan) and the results were calculated using Nikon software.

### Animal studies

The animal experiments were conducted in accordance with the approval of the Committee of our hospital. Four-week-old male BALB/c nude mice were employed as the experimental subjects. They received injections of PC3 cells (1 × 10^^6^) stably transfected with shCtrl or shRRM2. Once palpable tumors had developed, the animals were randomly divided into two groups (n = 5) and subjected to intraperitoneal injections of docetaxel (10 mg/kg) twice a week for a total of 3 weeks. Tumor measurements were taken every 2 days using calipers, and tumor volumes were calculated using the formula: V = 0.5 × length × width^^2^.At the conclusion of the study, we euthanized the mice and collected tumor samples.Regarding the castration of male mice before cell injection, we did not specify this procedure in our methods. Therefore, we want to clarify that the male mice in our study were not castrated prior to cell injection.

### Calculation of combination index

The synergistic effects analysis was conducted using the Calcusyn 2.0 program (CompuSyn software, Biosoft, Cambridge, United Kingdom). The program calculated the combination index (CI) values. The effects of the drug combination employed in this study were evaluated based on the CI derived from Chou-Talalay’s multidrug effect equation. A CI value less than 1 signifies synergistic effects, a value of 1 indicates additive effects, and a value greater than 1 suggests antagonistic effects.

### Statistical analysis

After at least three independent experiments, the results were averaged and the final data were measured as the mean ± standard deviation (SD). Two-tailed Student’s t-test was used for each index data and then one-way ANOVA, based on which Dunnett-test was applied to assess significance. A large number of clinical variables were collected in this study. For such data, the expanded Pearson chi-square test was mainly used, and the correlation analysis of the two groups used the classic Spearman’s correlation analysis. The overall survival time is the time from completion of surgery to follow-up. For the cumulative survival time recorded in this study, the Kaplan–Meier method is currently widely used, and the log-rank test was introduced between group comparisons to determine whether the difference was significant. For the adjusted risk ratio, this paper mainly evaluates with the help of multivariable Cox proportional risk model, on this basis, further obtain independent prognostic factors. All indicator data are entered into the latest version of the (SPSS) software (IBM, New York, USA) for processing. Taking 0.05 as the test level, P less than this value indicates a significant difference.

## Results

### Identification of prognostic cellular senescence-related DEGs in the TCGA cohort

**Fig. 1:**
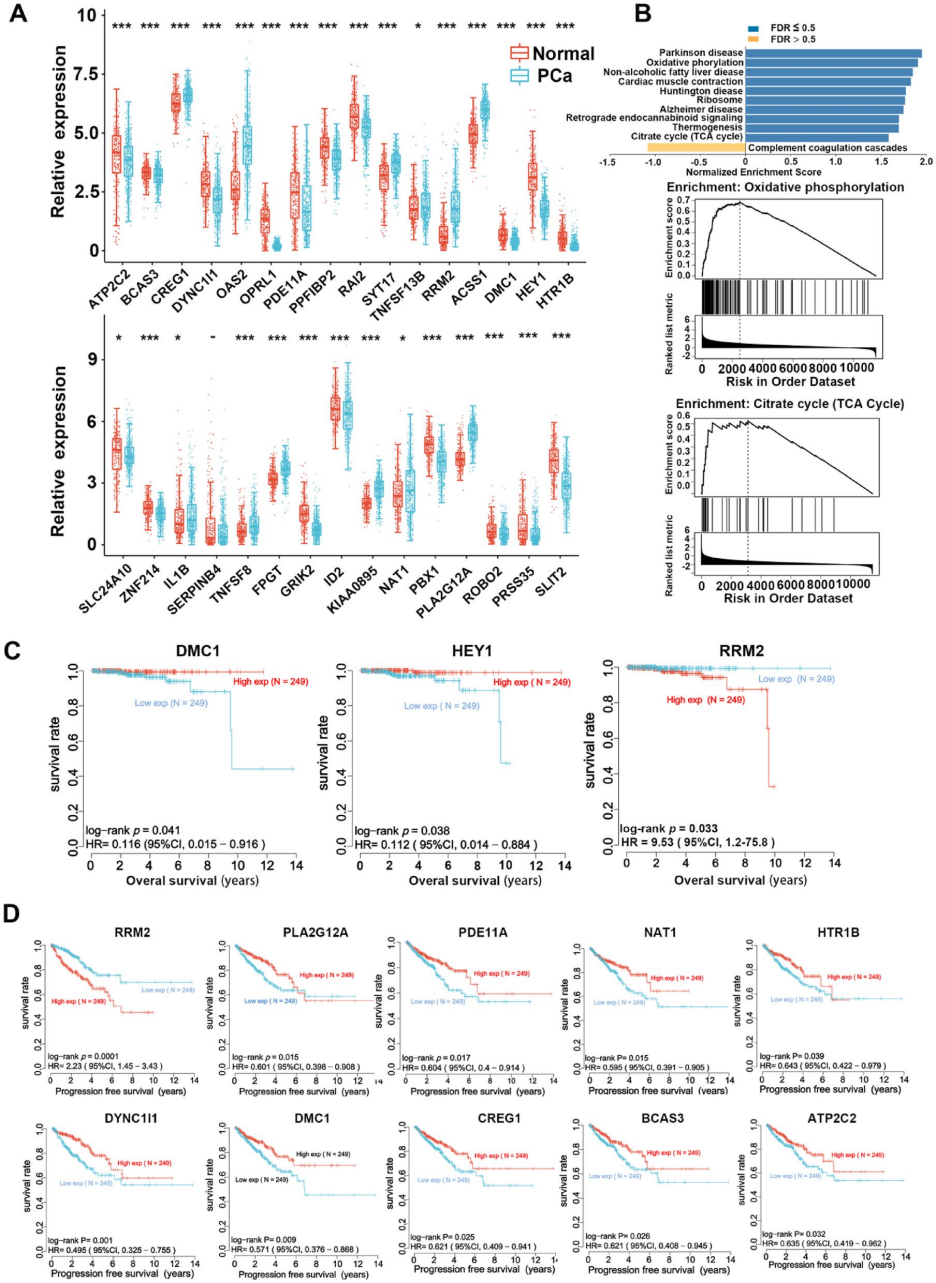
Analysis of cellular senescence-associated core genes in prostate cancer. **A** Differential expression analysis of cellular senescence-related genes (SRGs) between tumor tissues and adjacent non-tumor tissues. The heatmap displays the fold change in gene expression, with upregulated genes marked in red and downregulated genes marked in blue. **B** Gene Set Enrichment Analysis (GSEA) showing the enrichment of differentially expressed genes (DEGs) in pathways such as the (TCA cycle). The enrichment score and nominal *P*-value are provided. **C** Cox-regression analysis results demonstrating the association between three cellular SRGs and overall survival (OS) in prostate cancer (PCa) cases. The hazard ratio (HR) and *P*-value are shown. **D** Kaplan–Meier survival analysis illustrating the association between ten cellular aging-associated genes and progression-free survival (PFS) in PCa cases. The log-rank P-value and hazard ratio (HR) are provided

A total of 498 PCa patients from the TCGA-PRAD cohort and 297 normal samples from the TCGA-PRAD and GTEx cohorts were ultimately included in the study. We identified 34 cellular senescence-associated core genes from previously published literature [28], excluding undetected genes (GUCY1B1), pseudogenes (WTAPP1),and noncoding RNAs (C1ORF147),leaving 31 cellular senescence-associated core genes for further analysis.Most of the cellular senescence-related genes (SRGs) (30/31, 96.78%) were differentially expressed between tumour tissues and adjacent nontumor tissues (Fig. 1A). Three of the SRGs were correlated with OS and ten were correlated with PFS in the Cox regression analysis (Fig. 1C, D). In addition, 2 of these 3 OS-related genes (DMC1, RRM2) were also among the 10 PFS-related genes. Further GSEA suggested that the differentially expressed genes were enriched in oxidative phosphorylation, the citrate cycle (TCA cycle) and other pathways (Fig. 1B).

### Bioinformatics analysis of gene signatures related to RRM2 in PCa patients

RRM2 is an enzyme of significant importance in the processes of DNA synthesis and repair [29]. Its crucial role lies in facilitating the conversion of ribonucleotides into deoxyribonucleotides, fundamental components essential for DNA replication and repair mechanisms [30]. The dysregulation of RRM2 has been firmly associated with diverse cancer types, rendering it an appealing candidate for cancer therapeutic interventions. Moreover, it is worth noting that the inhibition of RRM2 has consistently demonstrated its ability to induce cell cycle arrest and promote apoptosis in cancer cells, thereby firmly establishing RRM2 as a highly promising therapeutic target [31-34] Additionally, the expression levels of RRM2 have emerged as valuable prognostic markers in numerous cancer types, underscoring their potential utility in predicting disease outcomes and guiding treatment decisions [35-37]. Furthermore, noteworthy findings reveal that RRM2 inhibition can sensitize cancer cells to chemotherapy and radiation therapy [34–36], indicating that RRM2 inhibition may have potential utility as a combination therapy with existing cancer treatments.

**Fig. 2:**
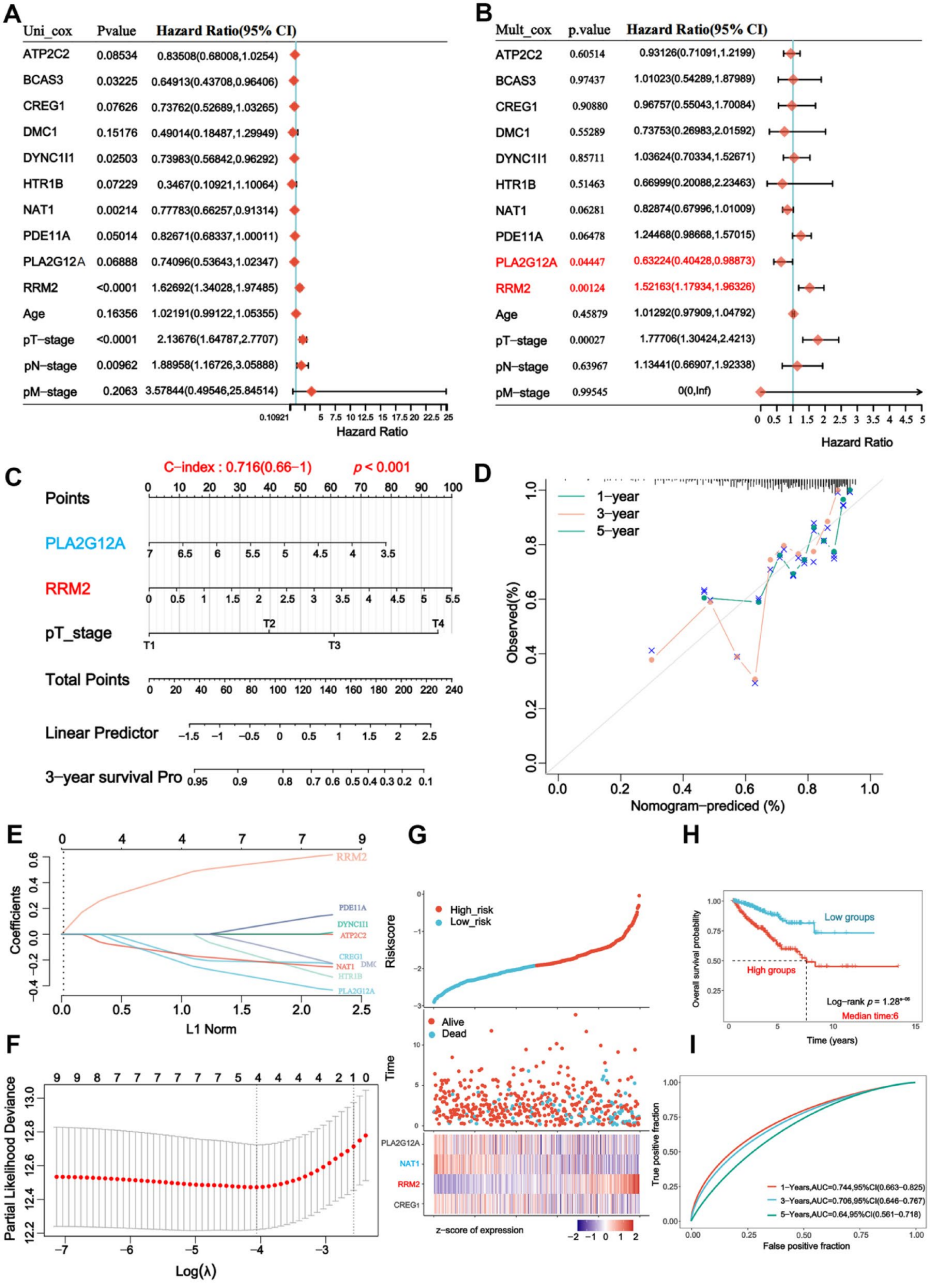
Prognostic Risk Analysis and Survival Outcomes in Prostate Cancer (PCa). **A**–**D** Cox regression analysis was performed to assess the prognostic significance of RRM2 in prostate cancer (PCa): **A** Results from the Cox regression analysis revealed that RRM2 expression is a key prognostic risk factor in PCa, indicating a statistically significant association with both (**B**) Overall Survival (OS) and (**C**) Progression-Free Survival (PFS). **D** The analysis demonstrated the strength of this association by displaying hazard ratios and their confidence intervals (CI), highlighting the impact of RRM2 expression on patient outcomes. **E**–**G** Survival analysis results indicated that high RRM2 level was obviously related to worse prognosis. **H** To further explore the prognostic value of RRM2, the cohort of PCa cases was stratified into high and low-risk groups using the median expression value of RRM2 as the threshold. This categorization yielded two distinct patient groups comprising (insert number here) individuals each, allowing for a more detailed analysis of RRM2’s impact on PCa prognosis. **I** The predictive accuracy of the risk score for Overall Survival (OS) was evaluated using a Receiver Operating Characteristic (ROC) curve analysis. This analysis assesses the sensitivity and specificity of the risk score in predicting survival outcomes, providing valuable insights into its clinical utility as a prognostic marker. The area under the ROC curve (AUC) was calculated to quantify the predictive performance of the risk score

Based on univariate and multivariate Cox-regression analysis, we identified RRM2 as a key prognostic risk factor in PCa (Fig. 2A–D). Additionally, we have established a corresponding prognostic model, leveraging the expression profiles of the aforementioned ten genes, employing classical Cox regression analysis. Following an extensive evaluation, we determined that an optimal threshold value of lambda yielded a predictive signature comprising four genes. Subsequent survival analysis unveiled that an elevated RRM2 expression mitigated the prognostic impact of the disease, as illustrated in Fig. 2E–G.

Presented below is the formulation of the prognostic risk model:

$${\text{Riskscore}}\, = \,\left( {0.{467}} \right)*{\text{RRM2}}\, + \,\left( { - 0.{171}} \right)*{\text{CREG1}}\, + \,\left( { - 0.{16}} \right)*{\text{NAT1}}\, + \,\left( { - 0.{2277}} \right)*{\text{PLA2G12A}} ({\text{lambda}}.{\text{min}}\, = \,0.0{172})$$

According to the median expression values, the patients were grouped, namely the high and low risk groups, with equal numbers in both groups. The results showed a close correlation between high risk and poor prognosis (Fig. 2H). For the OS risk score obtained in the early stage, the prediction performance is mainly evaluated by the time-dependent ROC curve, so that the (AUC) values are 0.744,0.706 and 0.64 in 1 year, 3 years and 5 years respectively. (Fig. 2I).

### RRM2 is upregulated in prostate cancer and associated with poor prognosis

**Fig. 3:**
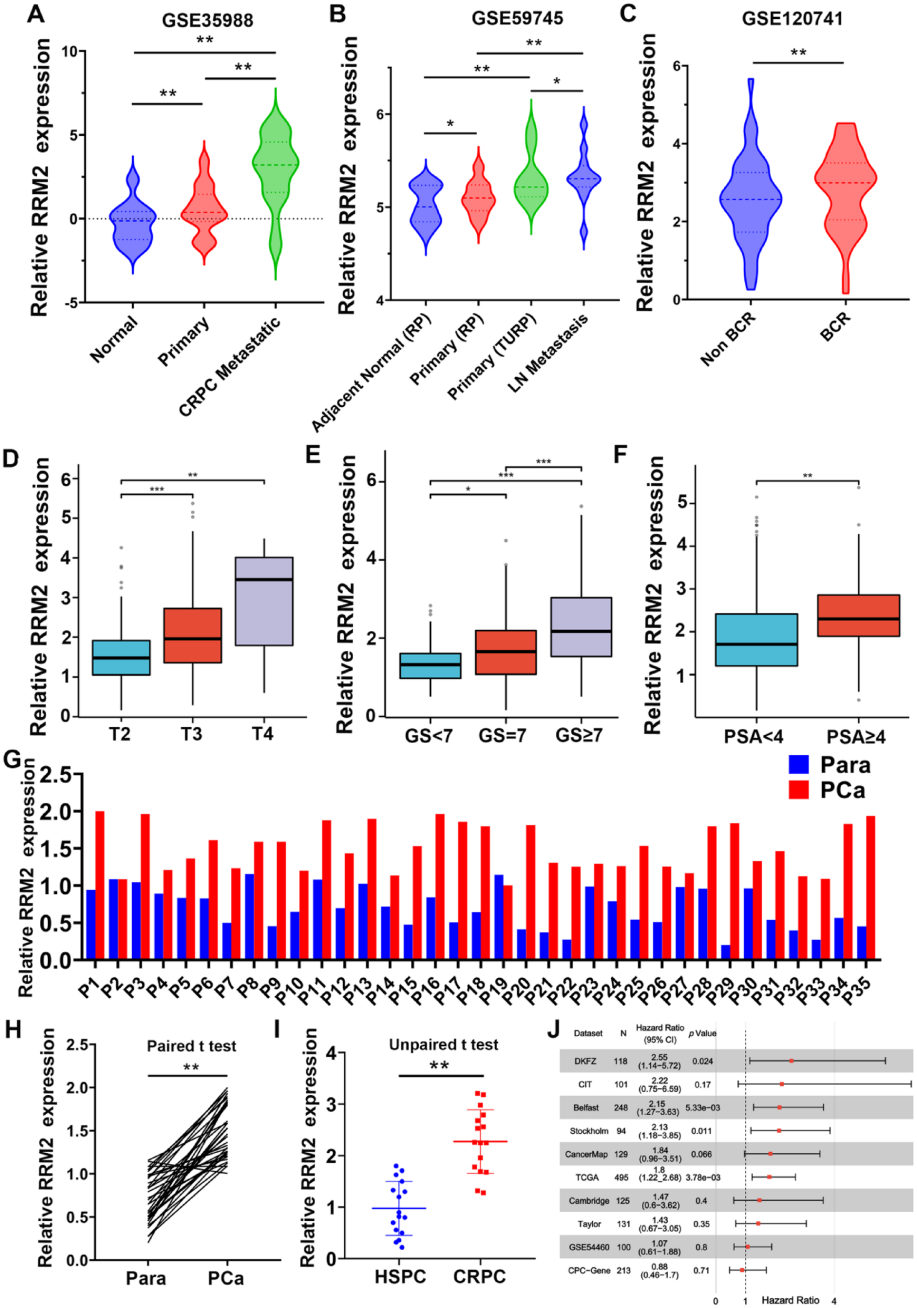
RRM2 is upregulated in prostate cancer and associated with poor prognosis. **A**, **B** Analysis of RRM2 mRNA expression levels in prostate cancer datasets from The Cancer Genome Atlas (TCGA) and Gene Expression Omnibus (GEO) databases. **C** Higher expression levels of RRM2 were observed in patients experiencing biochemical recurrence of prostate cancer (GSE120741). **D**–**F** Clinical data from The Cancer Genome Atlas database revealed a positive correlation between a high RRM2 expression level and a higher Gleason score, T stage, and serum PSA level, indicating an unfavourable clinical prognosis. **G**–**I** Higher expression levels of RRM2 were observed in prostate cancer tissue samples than in adjacent tissue samples. Additionally, RRM2 expression levels were significantly higher in castration-resistant prostate cancer (CRPC) tissues than in hormone-sensitive prostate cancer (HSPC) tissues. **J** Integrated analysis of multiple publicly available databases containing prognostic information showed that high expression of RRM2 indicated an unfavourable prognostic outcome in various survival cohorts

To elucidate the pivotal role of RRM2 in prostate cancer, we meticulously acquired relevant data from comprehensive sources, including the Cancer Genome Atlas (TCGA) database and other pertinent repositories, to investigate the expression levels of RRM2 mRNA across these datasets. The results are comprehensively presented in Fig. 3A, B. Notably, we observed a marked upregulation of RRM2 expression in prostate cancer tissues as compared to their healthy counterparts. Subsequent in-depth analyses revealed substantially elevated RRM2 levels in metastatic lesions (GSE35988, GSE59745), reinforcing its association with disease progression. Furthermore, we scrutinized patients experiencing biochemical recurrence of prostate cancer (GSE120741) and identified notably heightened RRM2 expression in these cases (Fig. 3C). Notably, our investigation based on clinical data from the TCGA database revealed a significant correlation between elevated RRM2 levels and higher Gleason scores and advanced T stage, indicative of an unfavorable clinical prognosis (Fig. 3D–F).

Similarly, in the clinical samples obtained from our research center, we observed elevated levels of RRM2 in prostate cancer tissue juxtaposed with lower expression levels in adjacent healthy tissue. A comparative analysis further underscored the heightened presence of this gene in castration-resistant prostate cancer (CRPC) tissue, while its expression was comparatively reduced in hormone-sensitive prostate cancer (HSPC) tissue. These findings align with the clinical data extracted from The Cancer Genome Atlas database, lending additional support to the observed patterns (Fig. 3G-I). Furthermore, we integrated and analysed multiple publicly available databases associated with prognostic data and found that high RRM2 level indicated worse prognosis in the survival cohorts (DKFZ, Belfast, Stockholm, TCGA) (Fig. 3J). This collective evidence underscores the pivotal role of RRM2 as a key regulatory molecule in the onset and progression of prostate cancer (PCA).

**Fig. 4:**
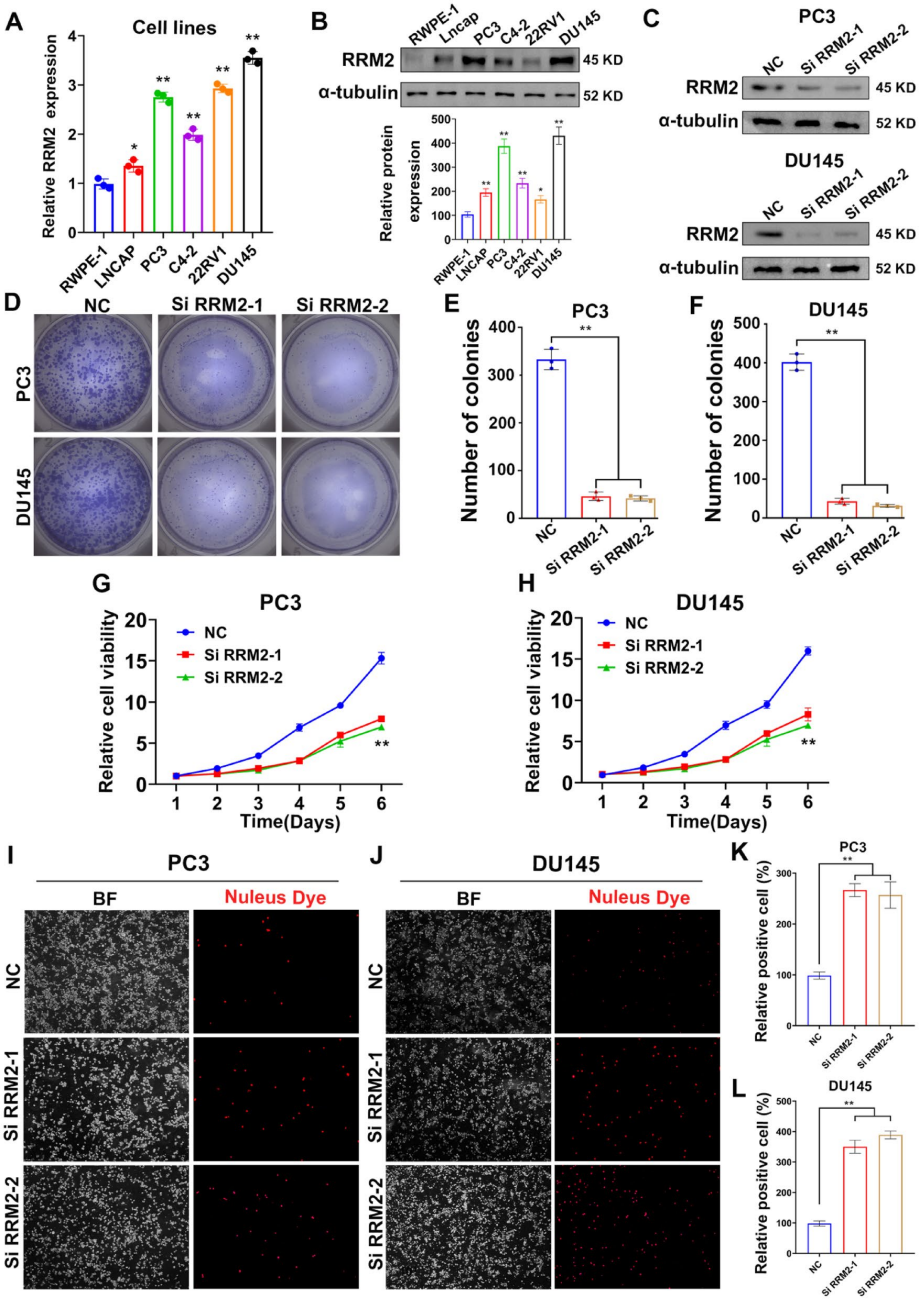
Functional analysis of RRM2 in prostate cancer progression. **A**, **B** Analysis of RRM2 mRNA and protein levels across different cell lines revealed heightened expression in PC3 and DU145 cells in comparison to RWPE-1 cell lines. **C** Effective downregulation of RRM2 in PC3 and DU145 cells, which originally exhibited elevated RRM2 expression, was achieved through the transfection of two independent siRNAs. **D**–**H** Notably diminished cloning and proliferation capacity of prostate cancer cells was observed consequent to RRM2 knockdown. **I**–**L** A significant increase in the rates of necroptosis and apoptosis observed in prostate cancer cells following RRM2 knockdown

To elucidate the roles of RRM2 in PCa progression, we first detected the expression of RRM2 in different PCa cells (Fig. 4A, B), to silence or regain the expression of RRM2 with higher efficiencies, as well as providing reliable conclusion through validating the function of RRM2 in two cell lines, we chose the two cell lines with moderate RRM2 expression, PC3 and DU145,to perform further functional assays and transfected two independent siRNAs to knockdown RRM2 in PCa cells (Fig. 4C). Our results unequivocally demonstrate that the suppression of RRM2 leads to a substantial reduction in the clonogenic and proliferative capacities of prostate cancer cells (Fig. 4D-H). Moreover, following RRM2 knockdown, we observed a notable increase in the rates of necroptosis and apoptosis in prostate cancer cells, as depicted in Fig. 4I-L and Additional file 1: Figure S1A–D. Furthermore, transwell assays revealed a significant inhibition in the migration speed and the number of migrated cells in RRM2-silenced PCa cells, as illustrated in Additional file 1: Figure S1E–J. In conclusion, the preliminary in vitro results show that RRM 2 aggravates the progression of prostate cancer, which provides a new direction for the treatment of the disease.

### RRM2 regulates sensitivity to docetaxel in prostate cancer cells

**Fig. 5:**
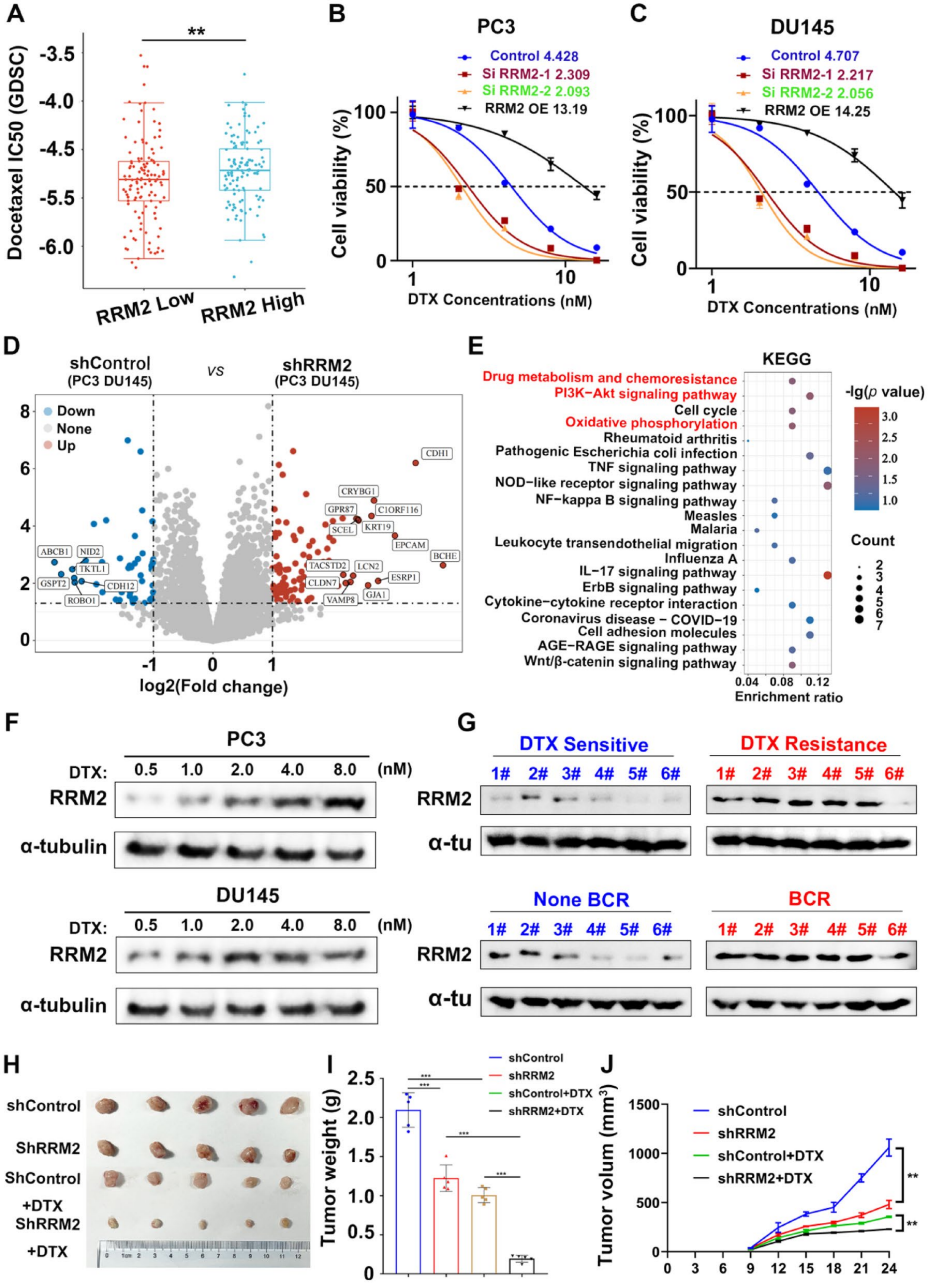
Mechanisms of RRM2-Mediated Docetaxel Resistance in Prostate Cancer. **A** Analysis in the GDSC database indicated that high level of RRM2 decrease the sensitivity of PCA to docetaxel therapy. **B**, **C** Drug screening assay in PC3 with RRM2 knockdown. The IC50 values of docetaxel were lower in RRM2 knockdown cells than in the control cells. **C** RNA-seq analysis in PC3 and DU145 cells after RRM2 knockdown. **D** RRM2 silencing inhibits the drug metabolism and chemoresistance pathway. **F** Increase in RRM2 translational expression level in PC3 and DU145 cells after docetaxel treatment in vitro. **G** Validation of the relationship between RRM2 expression at the translational level and clinical events in clinical samples. **H**, **I** RRM2 silencing enhanced the effects of docetaxel in vivo

RRM2 is a multifaceted factor in chemotherapy resistance, affecting DNA repair, cell survival, proliferation, and drug response [34–36]. Understanding its role in specific cancer types and contexts is essential for developing targeted therapies to overcome chemotherapy resistance. Analyses in the Cancer Drug Sensitivity Genomics (GDSC) database showed that high expression of RRM2 significantly weakened the sensitivity of cancer cells to docetaxel, a widely used chemotherapeutic agent (Fig. 5A). Drug screening experiments on PC3 and DU145 cells revealed that their RRM2 content was significantly higher than other prostate cancer cell lines. Further studies showed that the RRM2 knockdown cells had significantly lower resistance to docetaxel, compared to the controls (Fig. 5B, C). Further validation indicates a synergistic effect between RRM2 knockdown and docetaxel therapy (Additional file 1: Figure S3J, K). To better understand the fundamental principle of RRM2 regulating docetaxel sensitivity, two groups of cells (PC3 and DU145) were analyzed after RRM2 silencing using RNA-seq analysis (Fig. 5D). The results showed that RRM2 silencing significantly hindered drug metabolism and chemoresistance pathways, along with the oxidative phosphorylation pathway (Fig. 5E). Upon further investigation, an unexpected discovery emerged regarding the behavior of LNCaP and 22RV1 cells, initially exhibiting a low baseline expression of RRM2. These cells exhibited an increase in RRM2 expression in vitro following treatment with docetaxel and demonstrated a concentration-dependent trend within a specific treatment range (Additional file 1: Figure S3I). This intriguing finding was further validated in the PC3 and DU145 cell lines (Fig. 5F).Considering the evident association between RRM2 expression at the translational level and clinical outcomes, it can be inferred that this gene is notably more abundant in docetaxel-resistant prostate cancer tissues as opposed to sensitive tissues. Additionally, a heightened presence of this gene was observed in the diseased tissue of patients experiencing biochemical recurrence, in contrast to those without biochemical recurrence (Fig. 5G).Furthermore,our subsequent findings demonstrated that silencing RRM2 significantly potentiated the antitumor effectiveness of docetaxel in vivo (Fig. 5H, I), the calculated synergistic effect index was 0.732, affirming the presence of a synergistic effect between RRM2 knockdown and docetaxel treatment. Collectively, based on the insights gathered from the preceding discussions, it can be deduced that the ectopic expression of RRM2 plays a pivotal role in contributing to the development of docetaxel resistance in the clinical treatment of prostate cancer.

Additionally, we conducted preliminary investigations into the role of RRM2 in the process of docetaxel-induced senescence in prostate cancer cells. The results provide evidence that RRM2 promotes senescence in prostate cancer cells induced by docetaxel (Additional file 1: Figure S4).

### RRM2 interacts with ANXA1 to activate AKT signalling in prostate cancer cells

**Fig. 6:**
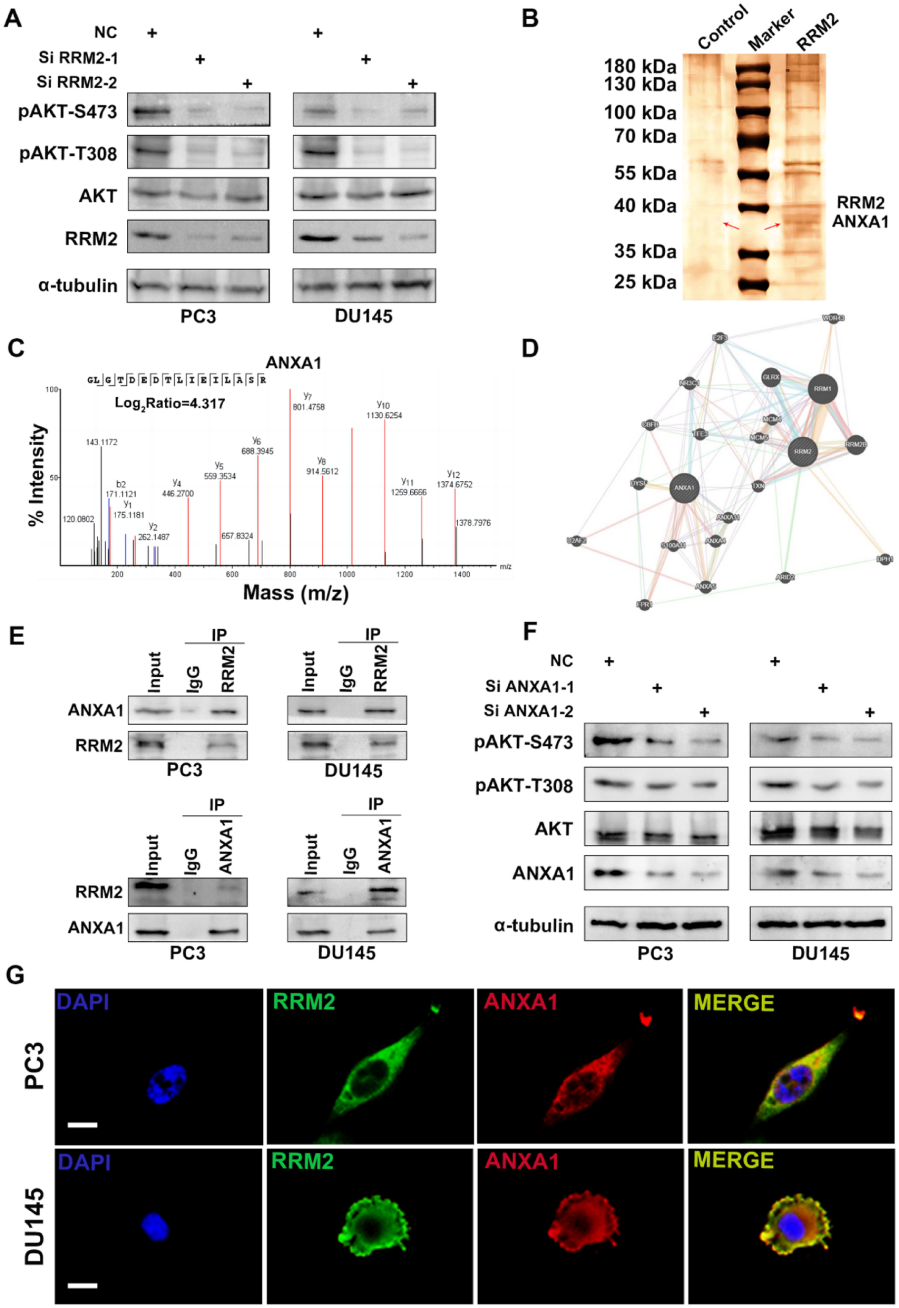
Mechanisms of RRM2-Mediated AKT Activation in Prostate Cancer. **A** RRM 2 knockdown somewhat reduced the phosphorylation level of AKT while improving the antitumor effect of docetaxel. **B**, **C** Immunoprecipitation, silver staining and mass spectrometry analysis shows that ANXA1 is a binding partner of RRM2 in PCa,and the Log_2_ ratio indicates the abundant presence of ANXA1 in the RRM2 immunoprecipitates. **D** Protein interaction network (PPI) analysis indicating the interaction relationship between RRM2 and ANXA1. **E** Coimmunoprecipitation (Co-IP) assay results confirming the interaction between RRM2 and ANXA1 in PC3 and DU145 cells. **F** ANXA1 silencing decreased the phosphorylation of AKT in both cell classes. **G** The expression level and colocalization of RRM2 and ANXA1 in PC3 and DU145 cells

Further study found that the reduction in RRM2 gene expression was found to be directly correlated with a decrease in AKT phosphorylation levels, ultimately leading to a heightened anti-tumor effect of docetaxel (Figs. 5 B, C; 6A). These experimental findings highlight the pivotal role of the PI3K/AKT signaling pathway in directly modulating docetaxel resistance in PCa. Furthermore, a noteworthy observation was made as pretreatment of various prostate cancer cells with docetaxel resulted in increased RRM2 expression.This gene’s potential regulatory influence on drug resistance appears to involve the activation of the AKT signaling pathway.

To elucidate the mechanism by which RRM2 activates AKT in diseased tissue, Immunoprecipitation, silver staining and mass spectrometry were employed to identify potential binding partners of this gene (Fig. 6B, C).The analysis revealed ANXA1 as the primary binding partner in body tissue, with the highest log_2_ratio. Protein interaction network (PPI) analysis further indicated an interaction between RRM2 and ANXA1 (Fig. 6D). Previous studies have suggested that ANXA1 promotes PI3K/AKT signaling by regulating FPR1 and FPR2 in cancerous cells [11, 14, 38]. Therefore, this study focuses on investigating whether RRM2 regulates AKT signaling through ANXA.

Co-immunoprecipitation (Co-IP) tests were conducted, demonstrating a close association between RRM2 and ANXA1 in both PC3 and DU145 cells (Fig. 6E). Silencing of ANXA1 differentially inhibited AKT phosphorylation in these cell lines (Fig. 6F). Subsequent fluorescence staining experiments confirmed the expression and co-localization of RRM2 and ANXA1 in both cell types (Fig. 6G). Based on the aforementioned discussion, it is speculated that ANXA1 plays a crucial role in the activation of AKT.

### RRM2 facilitated docetaxel resistance in PCa cells in an ANXA1-dependent manner

**Fig. 7:**
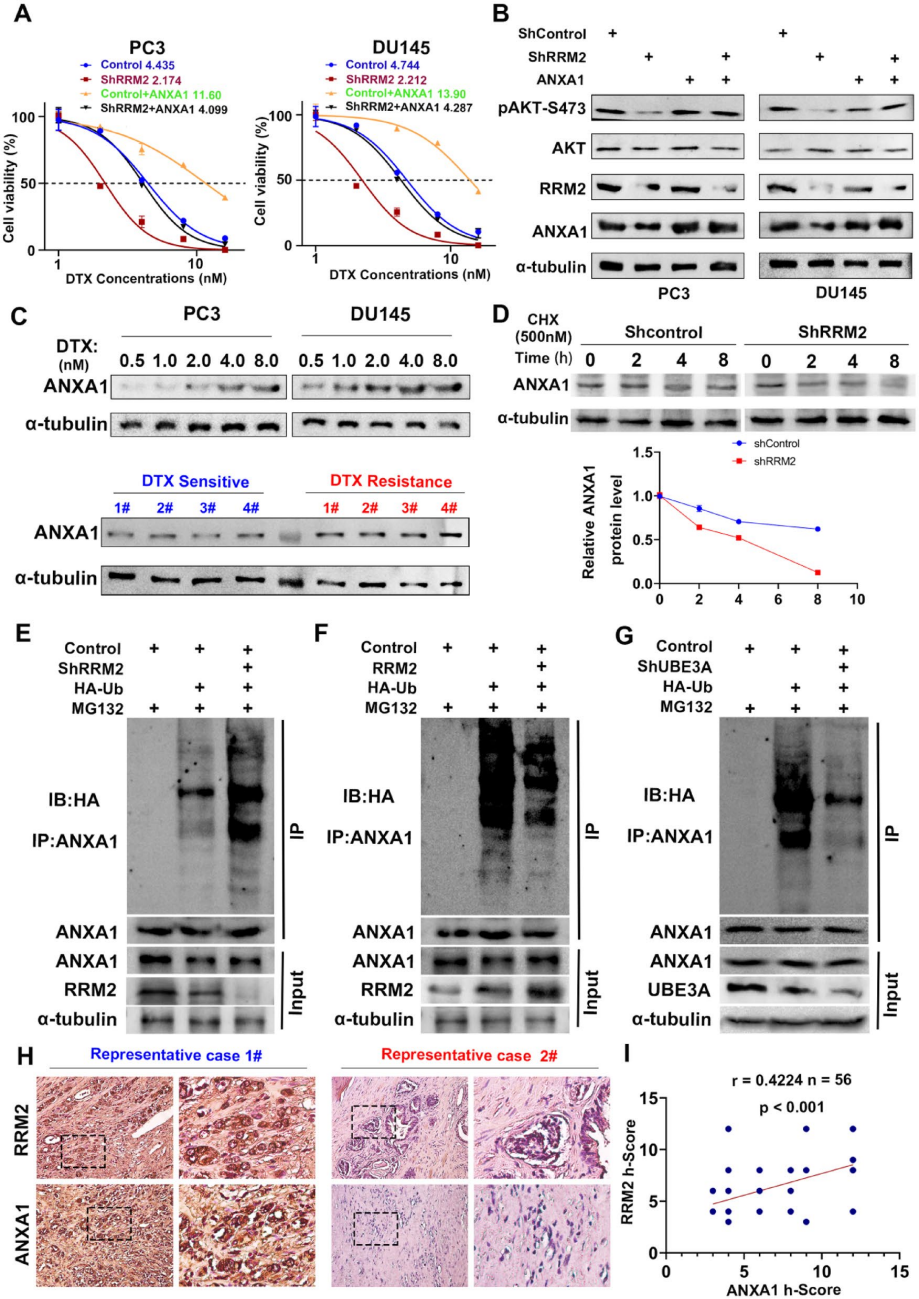
ANXA1 Mediates RRM2-Induced Activation of AKT and Docetaxel Resistance in Prostate Cancer. **A** RRM 2 knockdown significantly inhibited the phosphorylation of AKT in diseased tissue, while improving the therapeutic effect of docetaxel, and overexpression of ANXA 1 enhanced the phosphorylation of AKT. ANXA 1 silencing inhibited RRM 2 changes, which in turn affects AKT phosphorylation and drug sensitivity. **B** Expression of ANXA1 increases in LNCaP and 22Rv1 cells upon treatment with increasing concentrations of docetaxel within a certain range. ANXA1 level is obviously higher in docetaxel-resistant tumour group compared to that in sensitive group. **C** The docetaxel resistance induced by RRM2 suppression is largely reversed by ANXA1 overexpression in prostate cancer cells. **D**–**F** RRM2 knockdown decreases the protein level of ANXA1, shortens its half-life, and increases its ubiquitination level without affecting its mRNA level. **G** UBE3A knockout promotes the protein expression of ANXA1 in PC3 cells, which reflected that UBE3A plays a role in ANXA1 degradation. **H**–**I** The ANXA1 protein level is positively correlated with the RRM2 level in PCA tissues

ANXA1 was proven to be associated with drug resistance to promote cancer development [16, 39]. Silencing ANXA1 significantly reduced docetaxel resistance in PCa cells (Additional file 1: Figure S1L). Next, we overexpressed ANXA1 in RRM2-silenced PCa cells and observed that the decrease in docetaxel resistance resulting from RRM2 suppression was largely reversed by ANXA1 overexpression (Fig. 7A). Furthermore, RRM2 knockdown partially reduced AKT phosphorylation in PCa, leading to an improvement in the antitumor effect of docetaxel. Conversely, the increased expression of ANXA1 contributed to the restoration of AKT phosphorylation, there by enhancing docetaxel sensitivity (Fig. 7B, Additional file 1: Figure S1K). Building upon the aforementioned discussions, it is plausible to speculate that ANXA1 plays a pivotal role in RRM2-mediated AKT activation within the context of prostate cancer. Furthermore, our investigations revealed that PCa cells exhibited an increase in ANXA1 expression in vitro upon treatment with docetaxel, displaying a concentration-dependent trend within a specific treatment range. Additionally, ANXA1 expression was notably elevated in docetaxel-resistant samples and prostate cancer tissues experiencing biochemical recurrence (Fig. 7C, Additional file 1: Figure S3I).

To substantiate the hypothesis that RRM2 influences ANXA1 content in PCa cells, we meticulously examined the protein and mRNA levels of ANXA1 following RRM2 knockdown in prostate cancer cells. Intriguingly, a substantial reduction in ANXA1 protein levels was observed, while mRNA levels remained unaffected (Additional file 1: Figure S3O). Furthermore, it was noted that the half-life of ANXA1 considerably shortened following RRM2 gene knockdown, and ANXA1 ubiquitination levels showed a discernible increase during this process (Fig. 7D–F). Previous experimental studies have shown that the E3 ligase UBE3A interacts with the C-terminal domain of ANXA1, leading to ANXA1 degradation [11, 40]. Knockdown of RRM2 resulted in decreased ANXA1 protein level in PCa cells, and UBE3A knockout increased the protein levels of ANXA1 (Fig. 7G). Based on the above discussion, it can be inferred that RRM2 stabilizes ANXA1 in Pca tissue by competing with UBE3A. Furthermore, we found a close relationship between RRM2 content and ANXA1 in PCa tissue samples, with higher RRM2 content associated with increased ANXA1 levels (n = 56, P < 0.001) (Fig. 7H, I).

Drawing upon the clinical translational significance of RRM2, we proceeded to assess the impact of COH29, an RRM2 inhibitor, on PC3 and DU145 cells in vitro. The outcomes demonstrated that COH29 alone exhibited a noteworthy capacity to inhibit the growth of PC3 and DU145 cells. When administered in combination with docetaxel treatment, COH29 displayed a synergistic effect alongside the latter (Additional file 1: Figure S3A–F).

## Discussion

### RRM2 as a regulator of docetaxel sensitivity in prostate cancer

Our study provides compelling evidence for the role of RRM2 as a critical regulator of docetaxel sensitivity in prostate cancer. We observed a significant association between high RRM2 expression levels and docetaxel resistance in prostate cancer cells. Moreover, RRM2 knockdown resulted in increased sensitivity to docetaxel treatment, suggesting that targeting RRM2 could overcome resistance and enhance therapeutic efficacy.

The upregulation of RRM2 has been implicated in various cancer types and is associated with poor prognosis and resistance to chemotherapy [31, 41–43]. Our findings align with previous studies demonstrating the involvement of RRM2 in chemoresistance, further emphasizing its importance in prostate cancer progression and treatment response.

### Role of ANXA1 stabilization in RRM2-mediated docetaxel resistance

ANXA1, a protein involved in inflammation and apoptosis [31, 41–43], has been identified as a mediator of RRM2-induced sunitinib and PD-1 blockade resistance in renal cancer [11]. We observed that RRM2 knockdown led to decreased ANXA1 protein levels, suggesting that RRM2 stabilizes ANXA1 and promotes its expression in docetaxel-resistant cells.

The stabilization of ANXA1 by RRM2 may contribute to the development of resistance mechanisms in prostate cancer. ANXA1 has been implicated in various cellular processes, including drug resistance. It interacts with several signalling pathways, including the PI3K/AKT pathway, to regulate cell survival and the therapeutic response. A full understanding of the mechanisms underlying the interplay among RRM2, ANXA1, and the associated signalling pathways requires further investigation.

### Activation of the PI3K/AKT pathway in RRM2-mediated therapy resistance

The activation of the PI3K/AKT pathway has been widely implicated in cancer progression and resistance to therapy [45–47]. In our study, we observed a decrease in PI3K/AKT pathway activation following RRM2 knockdown in docetaxel-resistant prostate cancer cells. This finding suggests that the therapeutic resistance mediated by RRM2 may involve the activation of the PI3K/AKT pathway.

The PI3K/AKT pathway is known to play a critical role in various cellular processes, including cell survival, proliferation, and metabolism [48–50]. Dysregulation of this pathway is frequently observed in cancer and is associated with resistance to chemotherapy and targeted therapies. The involvement of the PI3K/AKT pathway in RRM2-mediated docetaxel resistance highlights the complex underlying mechanisms at play.

### Therapeutic potential of targeting RRM2 and the PI3K/AKT pathway in prostate cancer

Targeting RRM2 and the associated signaling pathways holds substantial promise as a therapeutic strategy to enhance the efficacy of docetaxel treatment in prostate cancer. Our study provides crucial insights into the potential of targeting RRM2, ANXA1, and the PI3K/AKT pathway to overcome docetaxel resistance and ultimately improve treatment outcomes.

Combination therapies that involve the co-administration of docetaxel with inhibitors targeting RRM2 or the PI3K/AKT pathway present an intriguing avenue to amplify treatment responses in prostate cancer. These combinatorial approaches may hold the key to overcoming resistance mechanisms and improving overall patient outcomes. Nonetheless, it’s important to emphasize that further preclinical and clinical studies are imperative to comprehensively delineate the optimal therapeutic strategies, potential side effects, and long-term outcomes associated with targeting RRM2 and the associated signaling pathways in the context of prostate cancer.

## Strengths and limitations

### Strengths

Comprehensive Investigation: Our study comprehensively examined the role of the senescence-related gene RRM2, the ANXA1 protein, and the PI3K/AKT pathway in regulating sensitivity to docetaxel therapy in prostate cancer. By investigating multiple molecular targets and their interactions, we provide a comprehensive understanding of the underlying mechanisms of therapeutic resistance.

Experimental Validation: We conducted extensive experimental validation using in vitro cell line models, including knockdown experiments and functional assays, to demonstrate the functional relevance of RRM2, ANXA1, and the PI3K/AKT pathway in docetaxel resistance. The related results increase the validity of our findings and support the biological significance of the observed associations.

Clinical Relevance: Our study contributes significantly to our understanding of the clinical relevance of RRM2 expression in prostate cancer. We observed a compelling association between RRM2 expression and docetaxel resistance, underscoring the potential of RRM2 as a predictive biomarker for therapeutic response. Notably, the observation of RRM2’s role in enhancing docetaxel resistance opens up possibilities for the development of tailored treatment strategies.Furthermore, we also explored the co-administration of the RRM2 inhibitor COH29 with docetaxel, revealing a synergistic effect. This suggests that combining COH29 with docetaxel in clinical settings may enhance treatment outcomes and mitigate resistance. Taken together, our findings have substantial implications for personalized treatment approaches in prostate cancer management, potentially leading to more effective therapies and improved patient selection based on RRM2 expression levels.

### Limitations

In Vitro Models: One limitation of our study is the predominant use of in vitro cell line models. While these models allow for controlled experiments, they may not fully represent the complexity of the tumour microenvironment and patient heterogeneity observed in clinical settings. Additional studies using in vivo models and patient-derived samples are needed to validate our findings and confirm their translational relevance.

Focus on Specific Pathways:This research focused on the role of RRM2, ANXA1, and the PI3K/AKT pathway in docetaxel resistance. However, resistance mechanisms in prostate cancer are multifactorial, and other molecular pathways may also contribute to therapeutic resistance. Therefore, our findings provide a limited view of the overall landscape of therapeutic resistance in prostate cancer.

Optimal Therapeutic Strategies: While we suggest targeting RRM2 and the PI3K/AKT pathway as potential therapeutic strategies, the optimal therapeutic approach and potential combination therapies require further investigation. Preclinical and clinical studies are needed to evaluate the safety, efficacy, and feasibility of specific inhibitors or modulators targeting these pathways.

Limited Sample Size: Our study was conducted with a specific cohort that may not represent the entire population of prostate cancer patients. Independent validation in larger cohorts with diverse patient populations is necessary to confirm the generalizability and reproducibility of our findings.

Clinical Relevance: Although we observed an association between RRM2 expression and docetaxel resistance, further investigation is needed to establish the clinical relevance of RRM2 as a predictive biomarker. Large-scale clinical studies incorporating comprehensive molecular profiling approaches and long-term follow-up are necessary to evaluate the prognostic and predictive value of RRM2 expression.

Addressing these limitations through future research endeavours will improve the understanding of the role of RRM2, ANXA1, and the PI3K/AKT pathway in therapeutic resistance in prostate cancer and facilitate the development of more effective treatment strategies.

## Conclusion

Our study provides novel insights into the key cellular senescence molecule RRM2 and its regulatory role in prostate cancer progression and resistance to docetaxel treatment. We demonstrate a positive association between RRM2 expression and docetaxel resistance and show the functional impact of RRM2 knockdown on increasing sensitivity to docetaxel. Furthermore, we elucidate the mechanistic role of RRM2 in stabilizing ANXA1 and activating the PI3K/AKT pathway, thus contributing to docetaxel resistance. Targeting RRM2, ANXA1, or the PI3K/AKT pathway may offer promising therapeutic strategies to overcome docetaxel resistance in prostate cancer. Our findings have implications for personalized treatment approaches and the development of predictive biomarkers to improve patient outcomes.

### Supplementary Information

**Additional file 1: Figure S1. A**–**D** Representative flow cytometric plots of the apoptosis assay and related statistical charts in prostate cancer cells following RRM2 knockdown. **E**–**J** Transwell assay images and quantitative data showing that RRM2 knockdown inhibited the invasion and migration of prostate cancer cells. **K** RRM2 knockdown and overexpression altered AKT phosphorylation and sensitivity to docetaxel in prostate cancer cells, and these effects were attenuated by ANXA1 silencing. **L** Silencing of ANXA1 inhibited the proliferation and reduced the docetaxel resistance of prostate cancer cells. P < 0.05, P < 0.01, **P < 0.001. **Figure S2. A**, **B** Representative fluorescence images showing the expression and colocalization of RRM2 and ANXA1 in PC3 and DU145 cells. **Figure S3. A**, **B** Cell viability of PC3 and DU145 cells under COH29 treatment. Cells were treated with indicated concentration of COH29 for 48 h and the viability was calculated by CCK8 assay. Data are showed as mean±SD of at three independent experiments. **C**–**F** The calculation of the synergistic effect index for COH29 and docetaxel therapy was performed using the Calcusyn 2.0 program. **G**, **H** Histogram showing knockdown efficacy of shRRM2 in PC3 and DU145 cells. **I** Increase in RRM2 translational expression level in LNCAP and 22RV1 cells after docetaxel treatment *in vitro*. **J**, **K** The synergistic effect index for RRM2-silenced and docetaxel therapy. **N** Elevation of ANXA1 translational expression levels in LNCAP cells following in vitro docetaxel treatment. **O** Histogram of mRNA levels of ANXA1 following RRM2 knockdown in PC3 and DU145 cells. **Fig. S4 A**–**C** Determination of β-Galactosidase Levels Following 24-Hour Pretreatment with Docetaxel in PC3 and LNCAP Cells, Along with Corresponding Quantitative Analysis Histogram. **D** The protein levels of γH2AX and H2AX were detected by Western blotting in Control, Si RRM2-1, Si RRM2-2 and pcDNA-RRM2 PCa cells. **H**–**K** ELISA Analysis of Senescence-Associated Secretory Phenotype (IL-6/IL-8) Following 24-Hour Pretreatment with Docetaxel in PC3 and LNCAP Cells. **Table S1. **The sequences of small interfering RNAs used in this study. **Table S2. **Antibodies used in this study. **Table S3 **The sequences of primers used in this study.

## Data Availability

All data will be provided upon reasonable request. All RNA-seq and mass spectrometry data will be uploaded to the public database after review.
