# Correction: The key cellular senescence related molecule RRM2 regulates prostate cancer progression and resistance to docetaxel treatment

**DOI:** 10.1186/s13578-023-01178-1

**Published:** 2024-02-01

**Authors:** Bisheng Cheng, Lingfeng Li, Yongxin Wu, Tianlong Luo, Chen Tang, Qiong Wang, Qianghua Zhou, Jilin Wu, Yiming Lai, Dingjun Zhu, Tao Du, Hai Huang

**Affiliations:** 1 Department of Urology, Sun Yat-Sen Memorial Hospital, Sun Yat-Sen University, Guangzhou, 510120 China; 2 Guangdong Provincial Key Laboratory of Malignant Tumor Epigenetics and Gene Regulation, Sun Yat-Sen Memorial Hospital, Sun Yat-Sen University, Guangzhou, 510120 China; 3 Guangdong Provincial Clinical Research Center for Urological Diseases, Sun Yat-Sen Memorial Hospital, Sun Yat-Sen University, Guangzhou, 510120 China; 4 Department of Urology, The Sixth Affiliated Hospital of Guangzhou Medical University, Qingyuan People?s Hospital, Qingyuan, Guangdong 511518 China; 5 Department of Urology, Nanfang Hospital, Southern Medical University, Guangzhou, 511430 China; 6 Department of Obstetrics and Gynecology, Sun Yat-Sen Memorial Hospital, Sun Yat-Sen University, Guangzhou, 510120 China

**Correction to: Cell & Bioscience (2023) 13:211**

10.1186/s13578-023-01157-6.

In this article [1], Tao Du should have been denoted as a corresponding author.

The original article has been corrected.
