# Supplementary material for: The key cellular senescence related molecule RRM2 regulates prostate cancer progression and resistance to docetaxel treatment
Source: Cell Biosci. 2023 Nov 15;13:211. doi: 10.1186/s13578-023-01157-6 (PMC10648385; doi:10.1186/s13578-023-01157-6)
Supplement: Supplementary file 1 — Additional file 1: Figure S1. A–D Representative flow cytometric plots of the apoptosis assay and related statistical charts in prostate cancer cells following RRM2 knockdown. E–J Transwell assay images and quantitative data showing that RRM2 knockdown inhibited the invasion and migration of prostate cancer cells. K RRM2 knockdown and overexpression altered AKT phosphorylation and sensitivity to docetaxel in prostate cancer cells, and these effects were attenuated by ANXA1 silencing. L Silencing of ANXA1 inhibited the proliferation and reduced the docetaxel resistance of prostate cancer cells. P < 0.05, P < 0.01, **P < 0.001. Figure S2. A, B Representative fluorescence images showing the expression and colocalization of RRM2 and ANXA1 in PC3 and DU145 cells. Figure S3. A, B Cell viability of PC3 and DU145 cells under COH29 treatment. Cells were treated with indicated concentration of COH29 for 48 h and the viability was calculated by CCK8 assay. Data are showed as mean±SD of at three independent experiments. C–F The calculation of the synergistic effect index for COH29 and docetaxel therapy was performed using the Calcusyn 2.0 program. G, H Histogram showing knockdown efficacy of shRRM2 in PC3 and DU145 cells. I Increase in RRM2 translational expression level in LNCAP and 22RV1 cells after docetaxel treatment in vitro. J, K The synergistic effect index for RRM2-silenced and docetaxel therapy. N Elevation of ANXA1 translational expression levels in LNCAP cells following in vitro docetaxel treatment. O Histogram of mRNA levels of ANXA1 following RRM2 knockdown in PC3 and DU145 cells. Fig. S4 A–C Determination of β-Galactosidase Levels Following 24-Hour Pretreatment with Docetaxel in PC3 and LNCAP Cells, Along with Corresponding Quantitative Analysis Histogram. D The protein levels of γH2AX and H2AX were detected by Western blotting in Control, Si RRM2-1, Si RRM2-2 and pcDNA-RRM2 PCa cells. H–K ELISA Analysis of Senescence-Associated Secretory Phenotype (IL-6/IL-8) Fol [file 13578_2023_1157_MOESM1_ESM.zip › New folder/Table S3.docx]

**Table S3 The sequences of primers used in this study.**

| **NO.** | **Target** | **Forward (5'-3')** | **Reverse (5'-3')** | |
| --- | --- | --- | --- | --- |
| **1** | **RRM2** | TGACCTGGTAGGAGCAGTAG | TTAGGGTTCTGCTCGTTTGC |  |
| **2** | **ANXA1** | CACTCTCCCTAGAGCGAGTG | TGCTTCAGCATAGCCATGAG |  |
| **3** | **GAPDH** | CAAGGCTGAGAACGGGAAG | TGAAGACGCCAGTGGACTC |  |
